# PD96 Are Continuous Noninvasive Blood Pressure Monitoring Devices Accurate Enough To Replace Invasive Monitoring? A Rapid Review

**DOI:** 10.1017/S026646232400343X

**Published:** 2025-01-07

**Authors:** Yin Zhien Tan, Keng Ho Pwee

## Abstract

**Introduction:**

The intra-arterial catheter (invasive method) is the clinical and reference standard for continuous blood pressure (BP) monitoring. Continuous noninvasive blood pressure (CNBP) monitoring methods, for example applanation tonometry, volume clamp, and cuffless BP monitoring devices are gaining popularity. This review clarified the evidence on the accuracy of CNBP monitoring devices, which could potentially replace invasive monitoring in clinical care.

**Methods:**

A systematic search was carried out to look for systematic reviews with the following elements:

- Population: patients needing continuous BP monitoring;
- Intervention: CNBP monitoring devices;
- Comparator: intra-arterial BP monitoring; and
- Outcomes: accuracy of BP monitoring.

The databases searched included PubMed (MEDLINE), Epistemonikos, and the Cochrane Database of Systematic Reviews. Two reviewers independently reviewed the search results and shortlisted relevant articles for retrieval of full texts. Included reviews were critically appraised with the AMSTAR 2 instrument and the findings were summarized in a narrative synthesis.

**Results:**

Three systematic reviews with meta-analyses of fairly good quality were included. The included primary studies were conducted in perioperative or critical care settings. Subgroup analyses by CNBP monitoring method were included in each meta-analysis. The findings from the systematic reviews were consistent. On average, CNBP devices consistently measured lower systolic BPs than the invasive method (mean difference <0) and measured higher diastolic BPs and mean arterial pressures than the invasive method (mean difference >0), with wide ranging 95 percent limits of agreement. It was evident from subgroup analyses that the measurements obtained from different CNBP methods varied significantly.

**Conclusions:**

This rapid review found the accuracy of CNBP monitoring devices to be suboptimal in comparison with invasive monitoring. CNBP devices should not be routinely used but may be considered when there is difficult arterial access, or in patients who do not require arterial puncture for other purposes.

